# Supplementary material for: Targeting Replication Stress Using CHK1 Inhibitor Promotes Innate and NKT Cell Immune Responses and Tumour Regression
Source: Cancers (Basel). 2021 Jul 25;13(15):3733. doi: 10.3390/cancers13153733 (PMC8345057; doi:10.3390/cancers13153733)

Proctor et al Supplementary Material

Methods

Annexin V flow cytometry

Following *in vitro* treatment of cells with CHK1i+LDHU or DMSO for 48 h, cells were harvested, washed and resuspended in Annexin V binding buffer (10 mM HEPES, 10 mM NaOH, 140 mM NaCl, 5 mM $CaCl_2$). Annexin V AF488 was added and incubated for 15 min, adding Propidium iodide (PI) for 5 min, then cells analyzed using a CytoFLEX S Flow Cytometer (Beckman Coulter, Lane Cove, Australia). Acquired data was analyzed using FlowJo software (TreeStar Inc., Ashland, OR, USA).

Figure Legends

Figure S1: A. The indicated melanoma tumoursphere lines were treated for 48 h with CHK1i 0.1 μM GDC-0575 + 0.1 mM HU then harvested and stained for Annexin V-FITC and propidium iodide (PI). The percentage of cells in each quadrant are shown. B. A2058 and SKMEL13 cells were grown in 96 well plates and treated for 2 days with either 0.1 μM GDC-0575 + 0.1 mM HU (GDC+HU), CHK1i 0.2 μM SRA737 + 0.1 mM HU (SRA+HU) with and without 100 μM zVAD-fmk. Cells were stained with the vital stains TMRE (mitochondrial viability) and H33342 (DNA) and Sytox Green to identify dead cells, and the cells imaged by high content imaging. TRME and Sytox Green staining showed that the two CHK1i GDC-0575 and SRA737 + 0.1 mM HU effectively killed the two cell lines, and zVAD effectively rescued the viability of the cells, but did not allow the cells to proliferate as did the controls.

Figure S2: The indicated human melanoma tumoursphere lines were treated with and without 0.2 μM SRA737 +0.1 mM HU (drugs) and at 48 h for FACS analysis of the cell surface expression of CD47 on live cells. This data is representative of two independent experiments.

Figure S3: A. Mouse melanoma lines YUMMUV1.7 and YUMMUV3.3 were treated with 0.1 mM HU and increasing doses of the CHK1i SRA737 for 72 h then assayed for viability using resazurin. The data are from triplicates. B. YUMMUV1.7 cells were treated with 0.2 μM SRA737 + 0.1 mM HU for 48 h then assayed for Annexin V-FITC and propidium iodide (PI) by flow cytometry.

Figure S4: A. C57BL/6J mice were immunised with YUMMUV3.3 cells treated *in vitro* with either 0.1 μM doxorubicin as a positive control for ICD or 0.2 μM SRA737+0.1 mM HU for 48 h. Freeze-thaw killed YUMMUV3.3 cells were used as a negative control for ICD and control injected mice as unimmunised mice. 12 days after immunisation mice were rechallenged with live YUMMUV3.3 cells into the opposite flank and tumour growth followed. N=5 mice each treatment. B. Kaplan Meier graph of the same experiment as A, showing time to tumour progression of the rechallenge tumours. Progression was scored when tumours exceeded 50 mm^3. C. Additional control injected mice, and the mice that were protected by immunisation with either SRA737+LDHU or doxorubicin treated YUMMUV3.3 injected cells from A., were rechallenged with live YUMMUV3.3 cells into the neck scruff at 60 days after the original immunisation and tumour growth assessed at day 24 and 27 after challenge.

Figure S5: A. Tumour growth of YUMMUV3.3 with or without CHK1i+LDHU treatment. B. The NanoString defined cell types and changes in population relative to controls. C. Images of CD8α staining and quantitation of CD8+ lymphocytes per 40X magnification field from 10 fields in three tumours collected at the end of experiment (day 24) for YUMMUV1.7 (Y1.7) and 3.3 (Y3.3) models. D. Three tumours for each treatment were harvested 2 days after final treatment and analysed using NanoString PanCancer Immune Profiling panel. Heat maps of the changes in expression compared to vehicle treated control of significantly altered genes are shown. The transcripts shown are all tumour associated on the basis of the transcript counts.

Figure S6: YUMMUV1.7 melanoma cells treated with or without CHK1i+LDHU for 24 h in vitro then immunoblotted for markers of A. NFκB activity, B. STING pathway. The change in band intensity relative to the control for each sample are indicated.

Figure S7: YUMMUV3.3 tumour bearing mice, either untreated or treated with CHK1i+LDHI were harvested at 16 day of treatment. The tumours were dissociated and the $CD45^{+}$ population analysed with either six marker panel of myeloid markers, or nine marker lymphoid set using flow cytometry. The data was subjected to unsupervised clustering of the major clusters for each and quantitated for 4-5 mice for each time point. A. The absolute numbers of each population (per million tumour cells) for the replicate mice. The heatmap of marker staining intensity of major clusters for the myeloid markers and cell types they specify present in Figure 6A were used here. B. The absolute numbers of each population (per million tumour cells) for the replicate mice. The heatmap of marker staining intensity of major clusters for the lymphoid markers and the cell types they specify used in Figure 6E

were used to define the cell types here. C. The percentage of cytokine staining T cells from the blood of the indicated mice at the indicated time points.

Figure S8: The individual tumour growth curves for the data shown in Figure 7A.

Figure S9: The individual tumour growth curves for the data shown in Figure 7B, C, A and C respectively. B. The percentage of cytokine staining T cells from the peripheral blood of the YUMMUV1.7 mice from two isotype control and three PD-1 antibody treated mice that had previously been treated with CHK1i+LDHU. The mice were harvested at the end of the experiment shown in A.

Figure S10: PD-L1 staining of sections of YUMMUV1.7 tumour from the experiment shown in Figure 6A, from control and SRA+LDHU treated mice at the time of ethical harvesting (when tumours reached $1cm^3$). These images are representative of three control and six treated mice.

Proctor et al Supp Figure S1

A

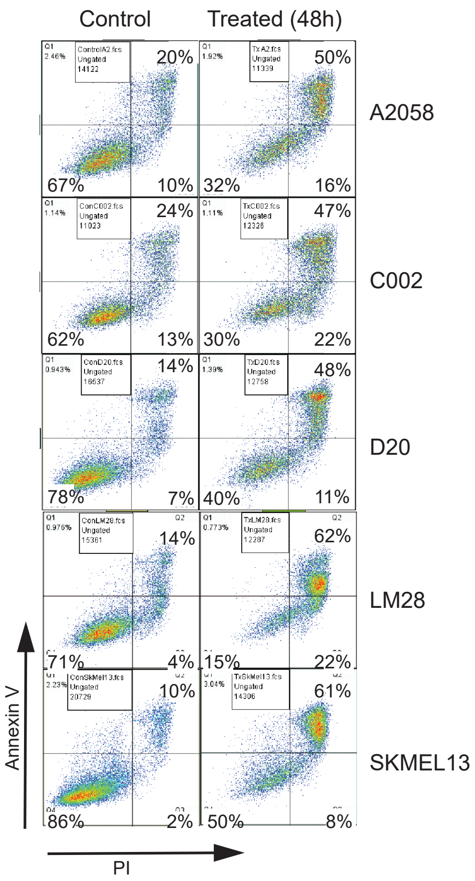

B

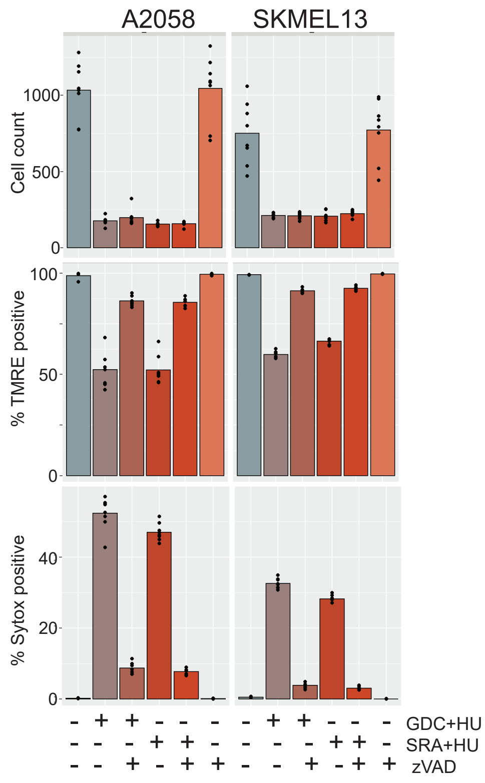

Proctor et al Supp Figure S2

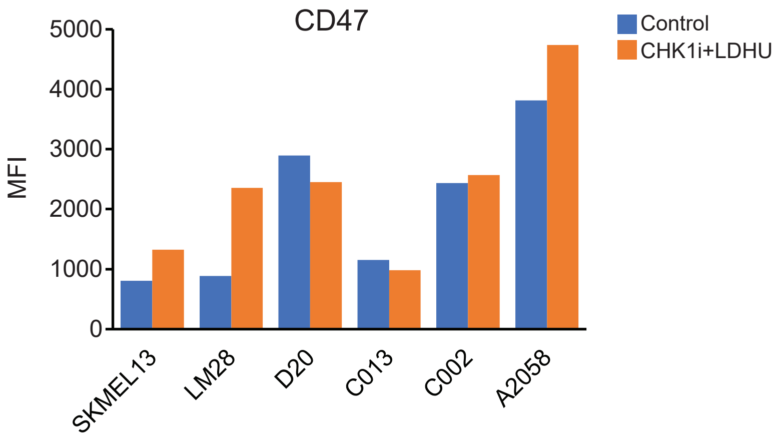

Proctor et al Supp Figure S3

A

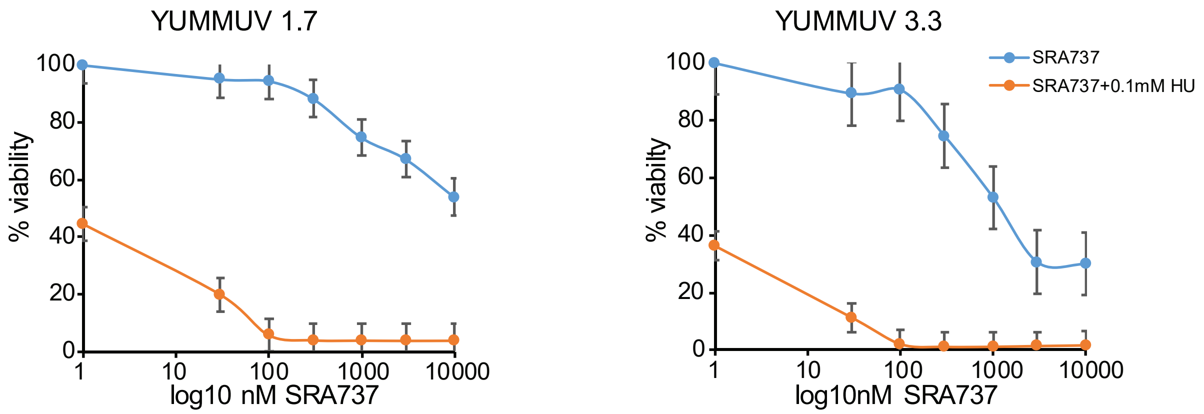

B

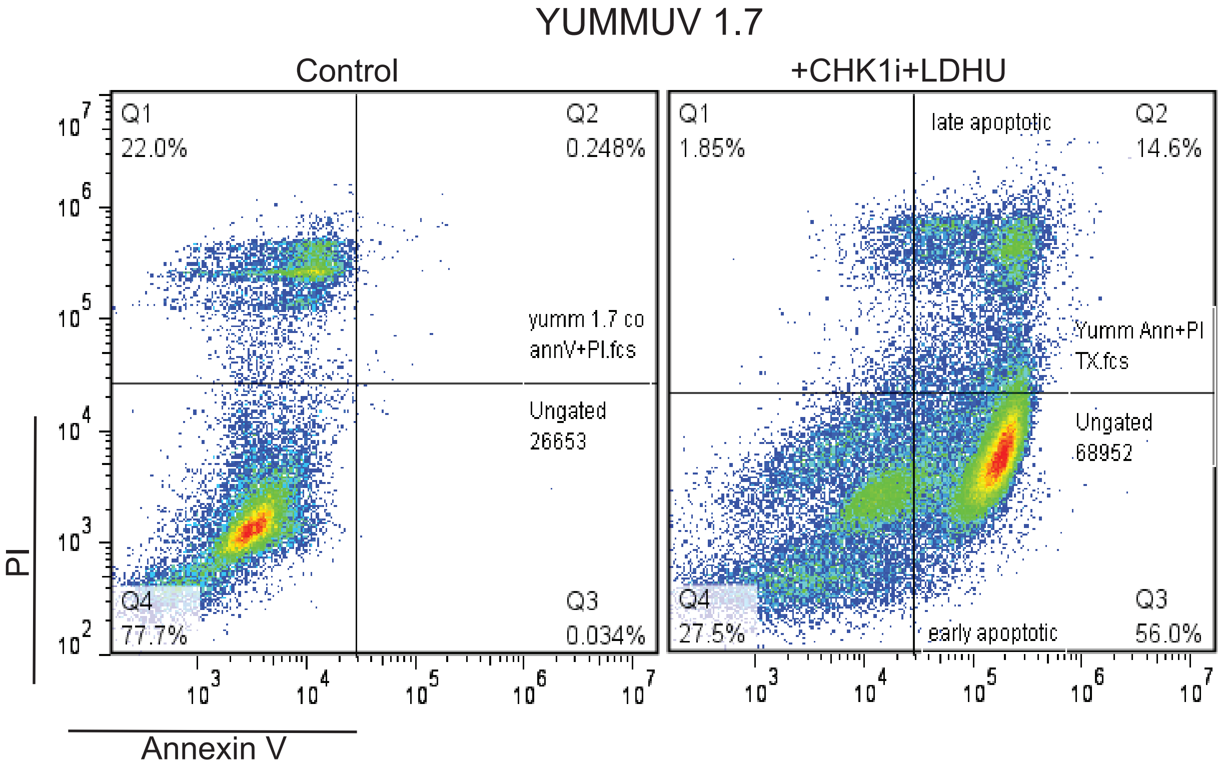

Proctor et al Supp Figure S4

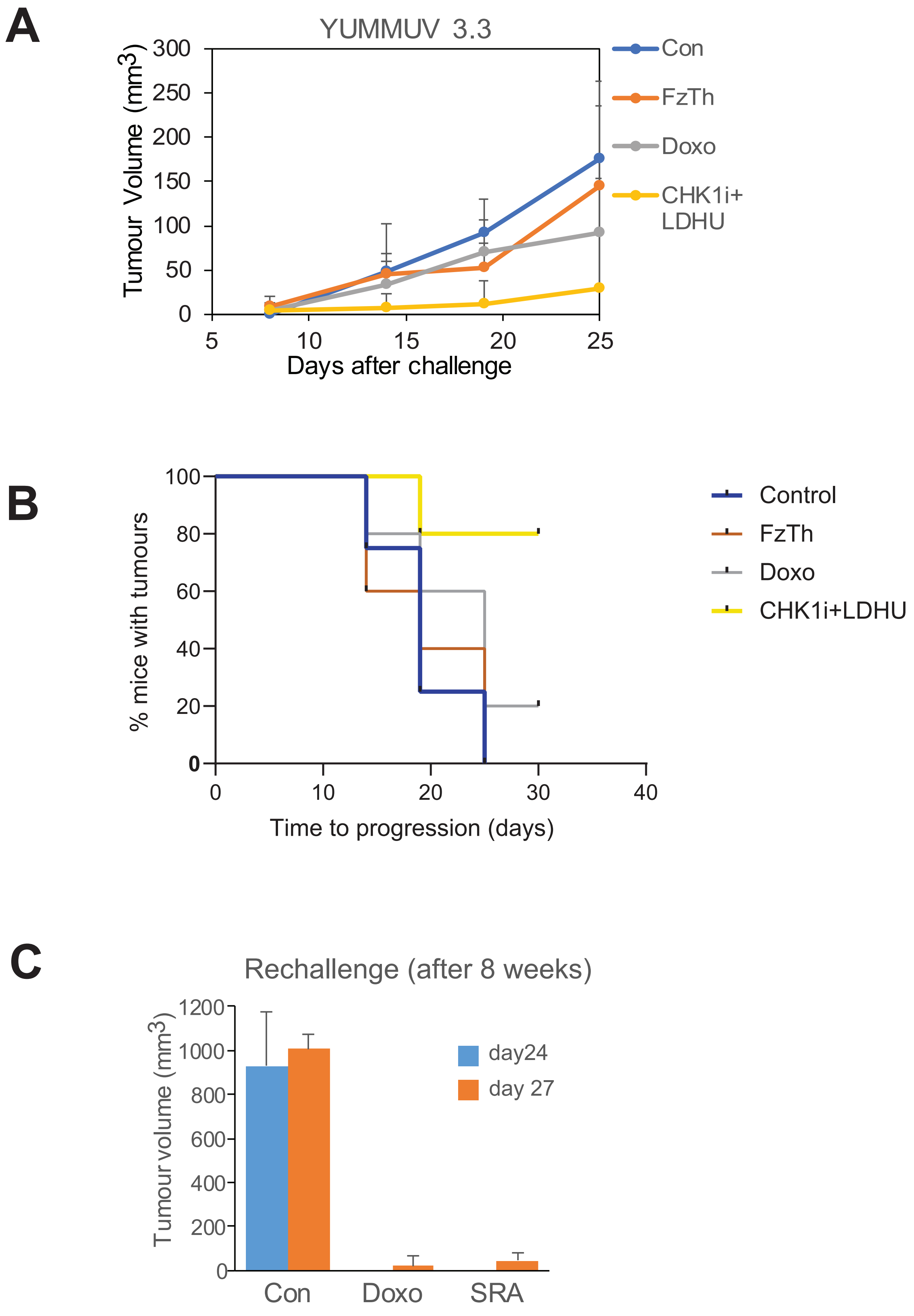

Proctor et al Supp Figure S5

A

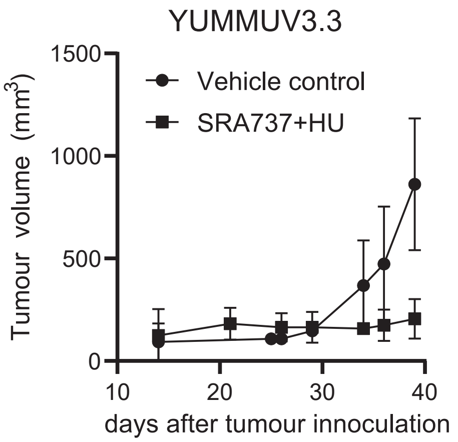

B

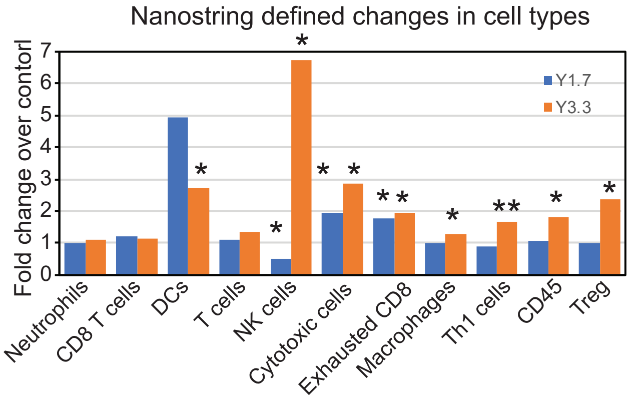

C

YUMMUV1.7 CD8α staining

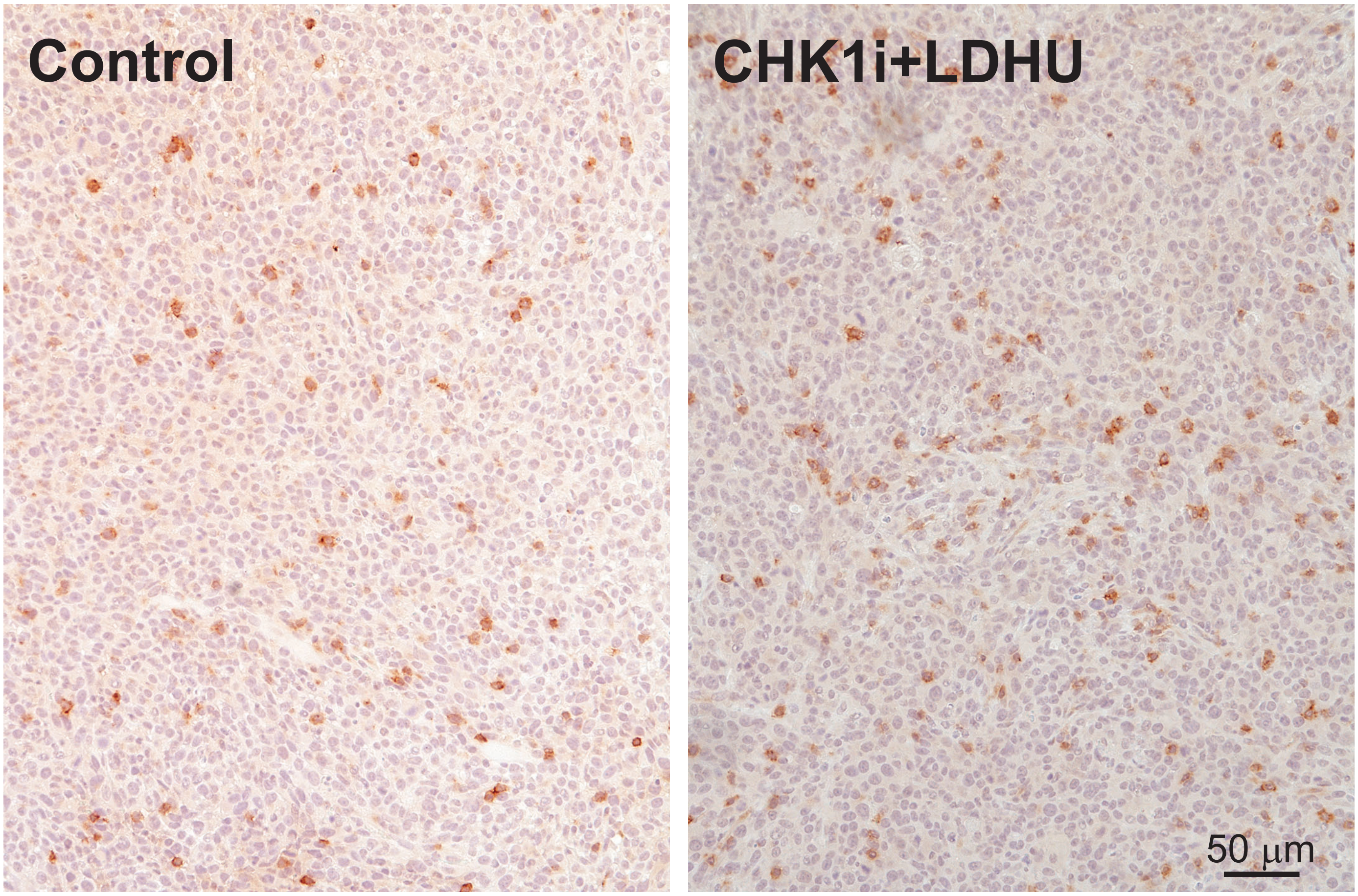

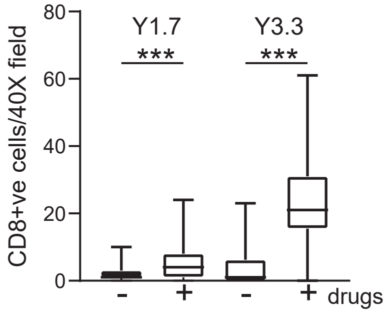

D

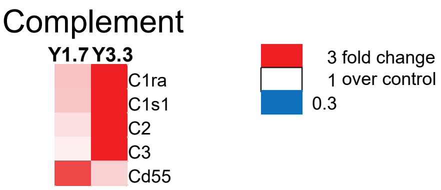

Proctor et al Supp Figure S6

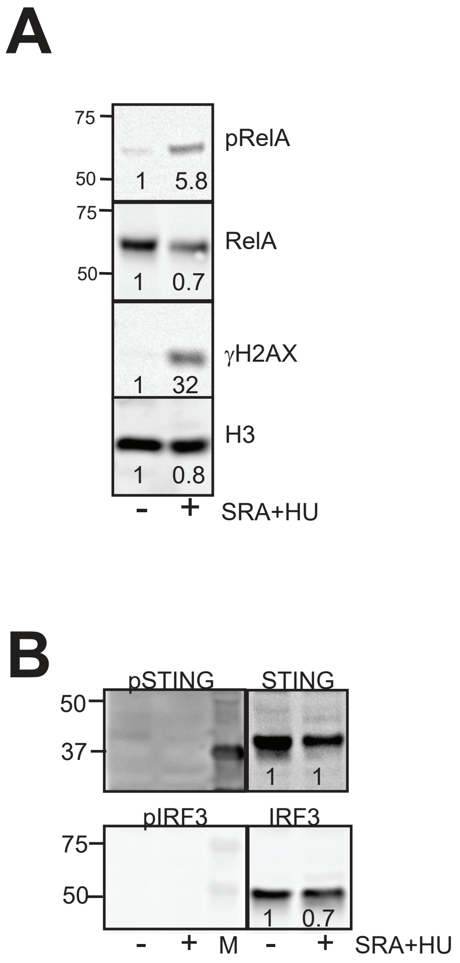

Proctor et al Supp Figure S7

A

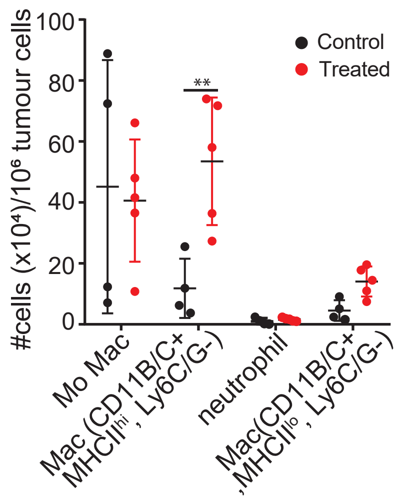

B

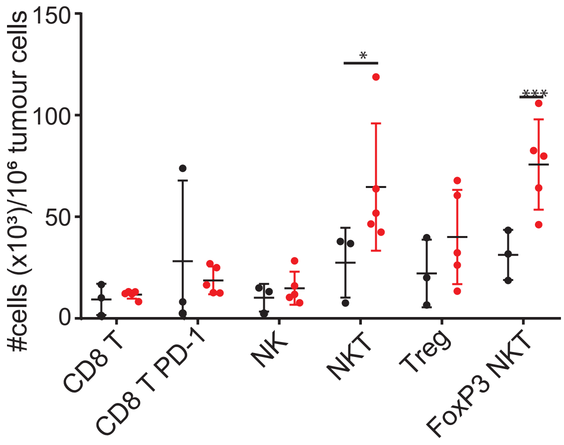

C

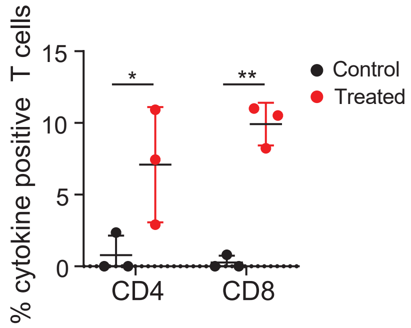

Proctor et al Supp Figure S8

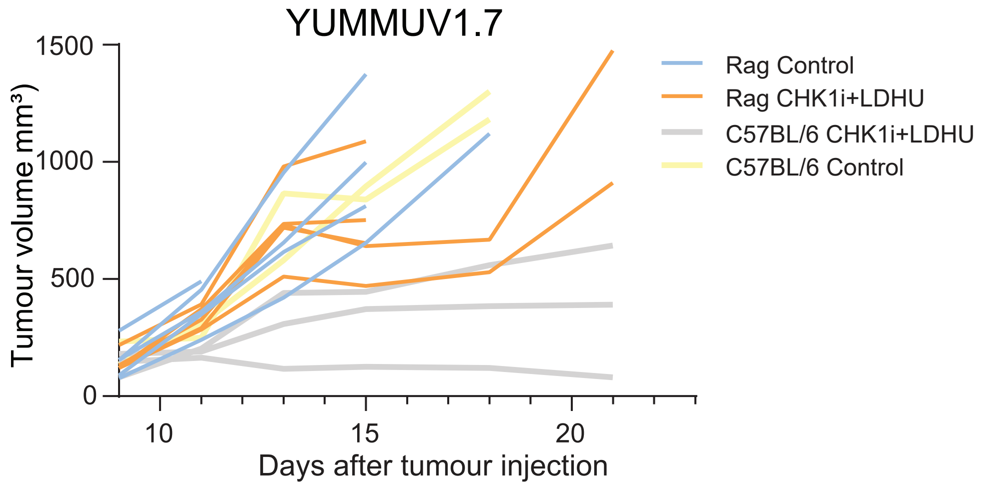

Proctor et al Supp Figure S9

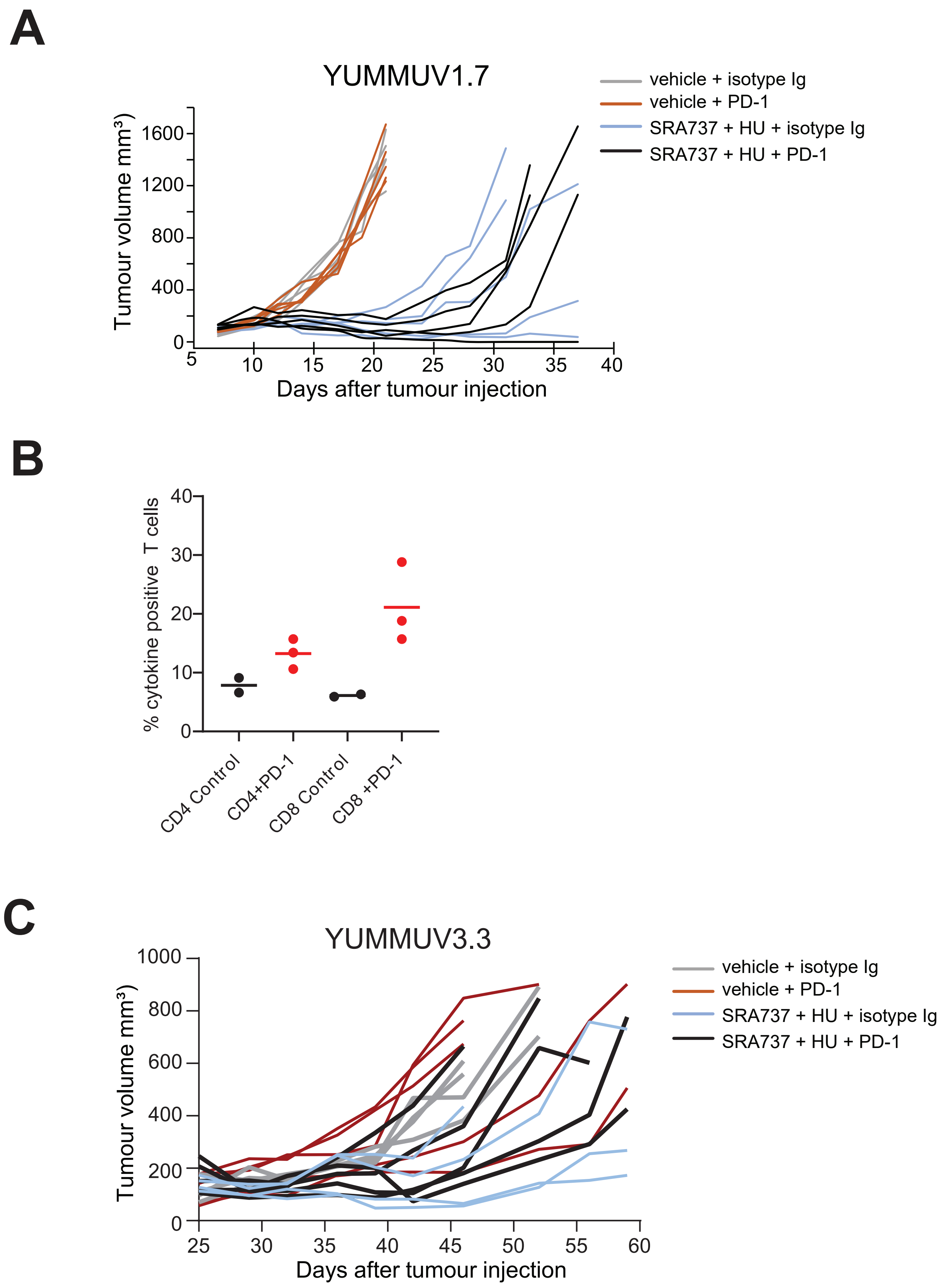

Proctor et al Supp Figure S10

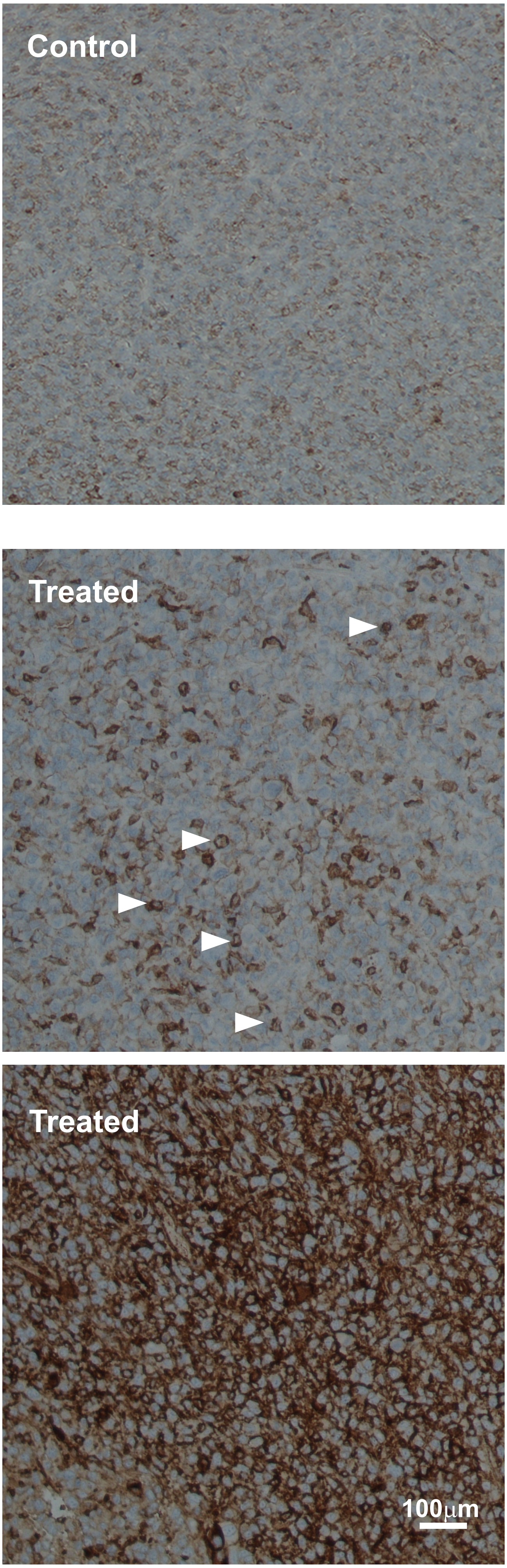

Figure 1

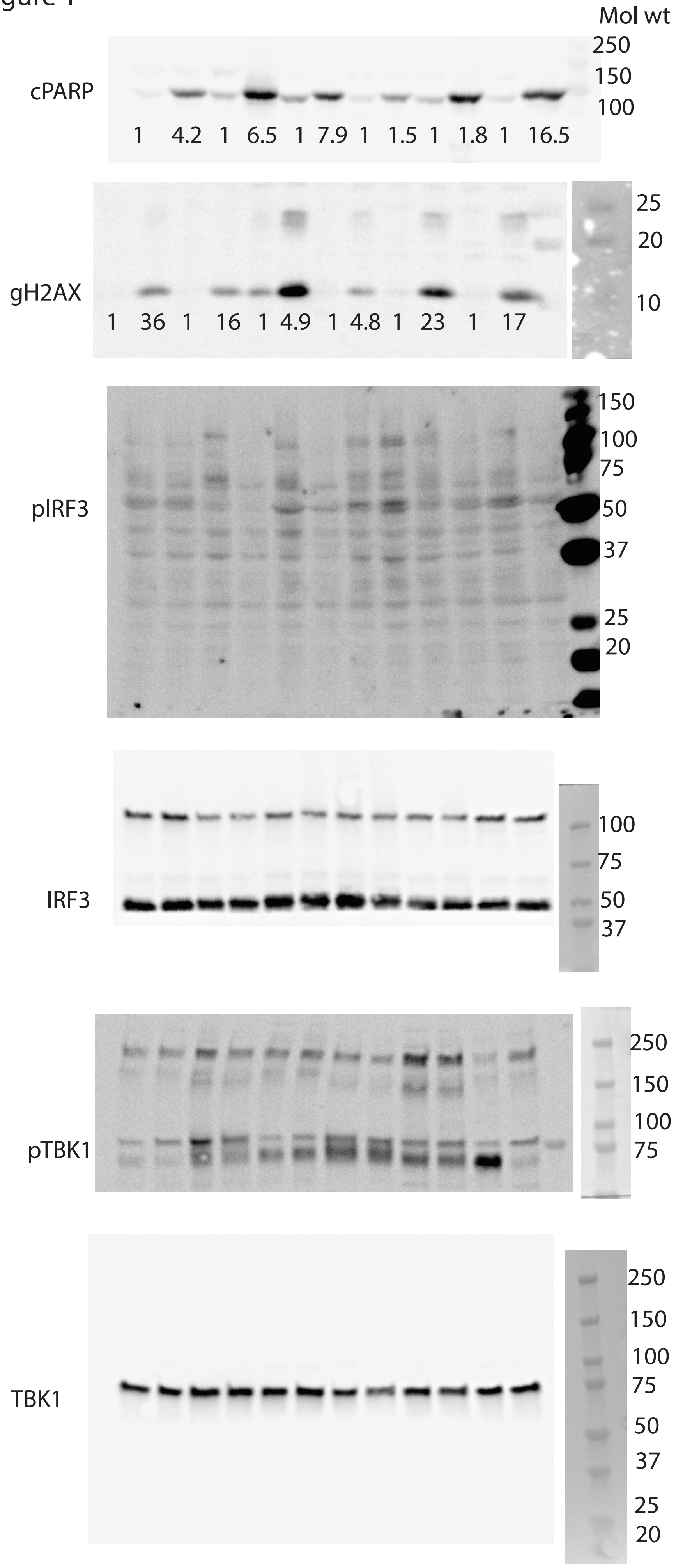
Mol wt
cPARP
250
150
100
1 4.2 1 6.5 1 7.9 1 1.5 1 1.8 1 16.5
gH2AX
25
20
10
1 36 1 16 1 4.9 1 4.8 1 23 1 17
pIRF3
150
100
75
50
37
25
20
IRF3
100
75
50
37
pTBK1
250
150
100
75
TBK1
250
150
100
75
50
37
25
20

Figure 1

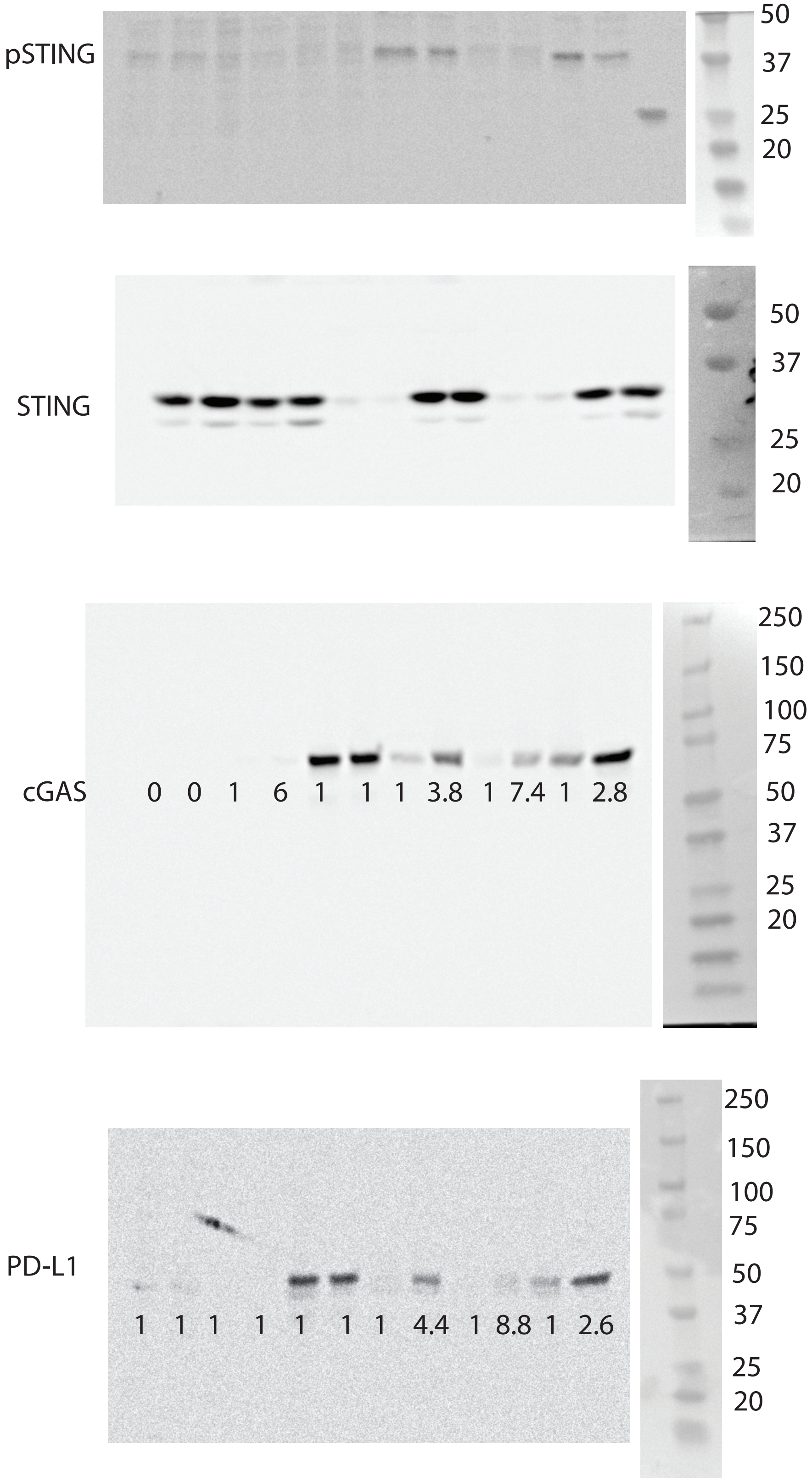
pSTING
50
37
25
20
STING
50
37
25
20
cGAS
0 0 1 6 1 1 1 3.8 1 7.4 1 2.8
250
150
100
75
50
37
25
20
PD-L1
1 1 1 1 1 1 1 4.4 1 8.8 1 2.6
250
150
100
75
50
37
25
20

crude Figure S6

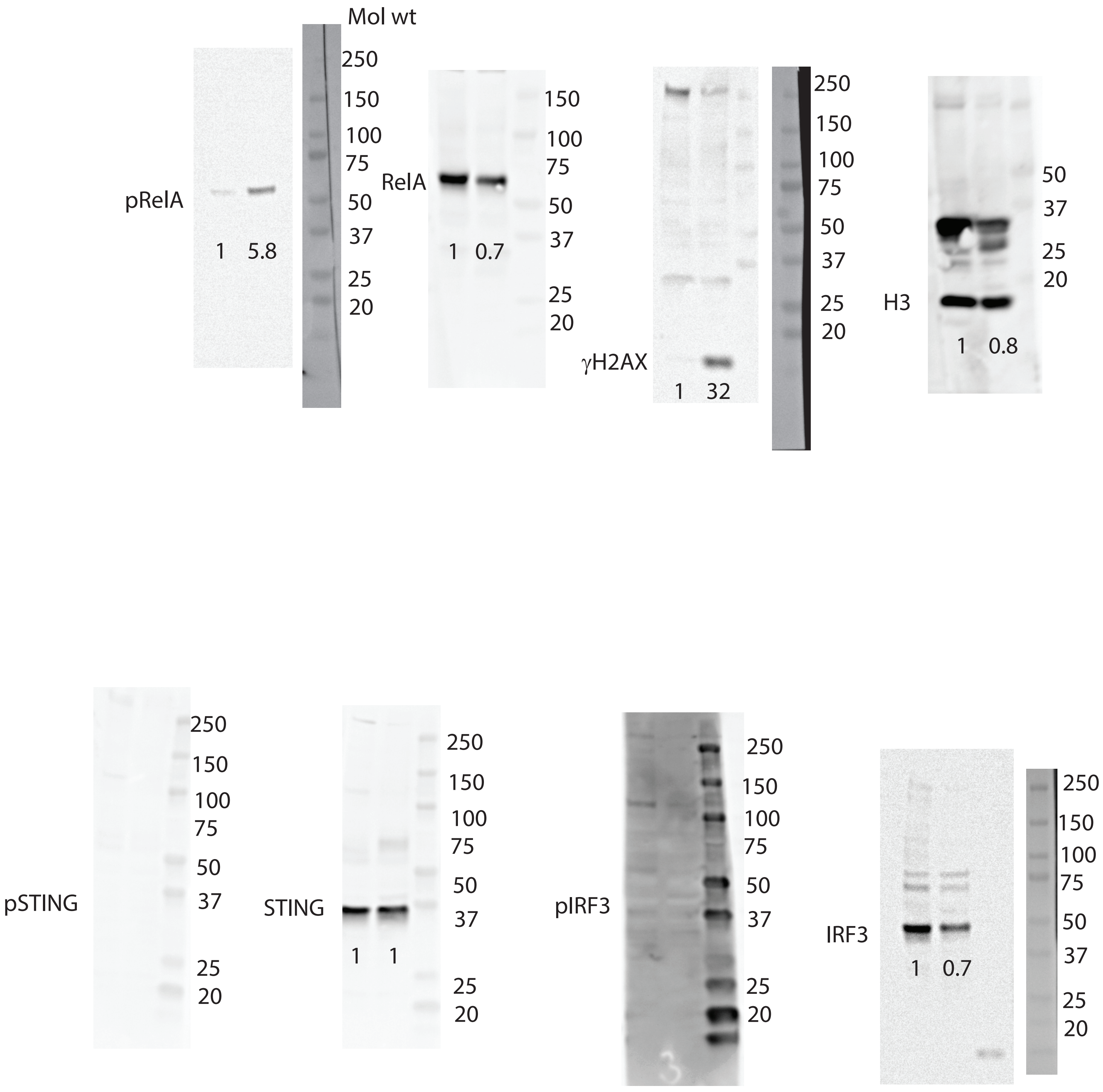

Supplement: Supplementary file 1 [file cancers-13-03733-s001.zip › cancers-1317539-supplementary.pdf]
